# Search for mesophotic octocorals (Cnidaria, Anthozoa) and their phylogeny: I. A new sclerite-free genus from Eilat, northern Red Sea

**DOI:** 10.3897/zookeys.680.12727

**Published:** 2017-06-14

**Authors:** Yehuda Benayahu, Catherine S. McFadden, Erez Shoham

**Affiliations:** 1 School of Zoology, George S. Wise Faculty of Life Sciences, Tel Aviv University, Ramat Aviv, 69978, Israel; 2 Department of Biology, Harvey Mudd College, Claremont, CA 91711-5990, USA

**Keywords:** Octocorallia, new genus, taxonomy, mesophotic coral ecosystem, Eilat, Red Sea

## Abstract

This communication describes a new octocoral, *Altumia delicata* **gen. n. & sp. n.** (Octocorallia: Clavulariidae), from mesophotic reefs of Eilat (northern Gulf of Aqaba, Red Sea). This species lives on dead antipatharian colonies and on artificial substrates. It has been recorded from deeper than 60 m down to 140 m and is thus considered to be a lower mesophotic octocoral. It has no sclerites and features no symbiotic zooxanthellae. The new genus is compared to other known sclerite-free octocorals. Molecular phylogenetic analyses place it in a clade with members of families Clavulariidae and Acanthoaxiidae, and for now we assign it to the former, based on colony morphology. The polyphyletic family Clavulariidae is, however, in need of a thorough revision once the morphological distinctions among its phylogenetically distinct clades are better understood.

## Introduction

Sclerites are microscopic, calcitic skeletal elements embedded in the tissues of certain groups of invertebrates, such as holothurians, tunicates and octocorals. Amongst the latter, they are considered to be one of the most prominent characteristic features and their form and anatomical arrangement are of major taxonomic importance (Fabricius and Alderslade 2001). Octocoral sclerites may vary greatly in morphology and size among different parts within a colony as well as among distinct taxa. They also differ in density and spatial orientation in the coenenchyme, and therefore in the degree to which they provide mechanical support and protection to colonies. In some taxa (e.g. gorgonians belonging to the subordinal group Scleraxonia) dense aggregations of fused sclerites form rigid axes that support very large, arborescent colonies (Fabricius and Alderslade 2001). In other taxa (e.g. some Nephtheidae) there is no skeletal axis and relatively few sclerites can be found in the coenenchyme, but bundles of large sclerites nonetheless support and protect individual polyps. Rarely, octocoral species may lack sclerites entirely.

Octocorals that completely lack sclerites or other calcitic skeletal elements have been described previously within seven octocoral families. These include the Acanthoaxiidae: *Acanthoaxis wirtzi* Ofwegen & McFadden, 2010; Acrossotidae: *Acrossota amboinensis* (Burchardt, 1902) (see also Alderslade and McFadden 2007); Alcyoniidae: *Malacacanthus capensis* (Hickson, 1900) (see also Williams 1987, 1992); Clavulariidae: *Clavularia celebense* Hickson, 1894, *C. reptans* Hickson, 1894, *C. pregnans* Thomson & Henderson, 1906, and *Phenganax parrini* Alderslade & McFadden, 2011; Cornulariidae: *Cervera* spp. López-Gonzáles et al., 1995, *Cornularia cornucopiae* (Pallas, 1766) (see also López-González al. 1995) and *C. pabloi* McFadden & Ofwegen, 2012; Dendrobrachiidae: *Dendrobrachia* spp. (Opresko & Bayer, 1991); and Xeniidae: *Anthelia gracilis* (May, 1898); *Cespitularia coerulea* May, 1898; *C. taeniata* May, 1899; *C. wisharti* Hickson, 1931; *C. hypotentaculata* Roxas, 1933 and *C. quadriserta* Roxas, 1933; *Efflatounaria totoni* Gohar, 1939; *Heteroxenia ghardaqensis* Gohar, 1940; *Xenia quinqueserta* May, 1898; *X. tumbatuana* May, 1898; *Xenia hicksoni* Ashworth, 1899; *X. sansibariana* May, 1899; *X. kükenthali* Roxas, 1933; *X. fimbriata* Utinomi, 1955; *X. novaecaledoniae* Verseveldt, 1974 and *X. mucosa* Verseveldt & Tursch, 1979. So far, the families Acanthoaxiidae, Acrossotidae, Cornulariidae and Dendrobrachiidae include only sclerite-free species; it should, however, be noted that some of the original type-material of the Cornulariidae should be re-examined in order to confirm the status of their sclerites (see also López-González and Garcia-Gomez 1995).

The majority of species in the families Alcyoniidae, Clavulariidae and Xeniidae have sclerites, however species without sclerites represent unusual exceptions. The families Alcyoniidae and Clavulariidae are highly polyphyletic (McFadden and Ofwegen 2012, 2013), and the few species that lack sclerites belong to unique clades that are not closely related to other members of those families. The depth of collection has been indicated for some of the above sclerite-free species, but none specifically refer to mesophotic coral ecosystems (MCEs). Thus, these octocorals are principally shallow-reef inhabitants (<30 m depth), except for *Dendrobrachia* spp., which are deep-water inhabitants. A recent remote-operated vehicle survey (ROV) conducted in Eilat (Gulf of Aqaba, northern Red Sea) at mesophotic depths (see also Benayahu et al. 2017) revealed a sclerite-free octocoral, whose morphological features and phylogenetic affinities justify the establishment of a new genus, described below.

## Materials and methods

Samples were collected by ROV (ECA H800) operated at a depth range of 50-190 m, by Sam Rothberg R/V of the Interuniversity Institute for Marine Sciences in Eilat (IUI). *In situ* photography was carried out by a low-light black and white camera VS300 (Eca Robotics) and 1CAM Alpha HD camera (SubCimaging). Samples were obtained by the ROV arm; fragments were removed on board and preserved in 100% ethanol for molecular work. The original samples were placed in 70% ethanol for taxonomic identification, and deposited at the Steinhardt Museum of Natural History, Israel National Center for Biodiversity Studies (ZMTAU).

### Molecular phylogenetic analyses

DNA was extracted from EtOH-preserved samples, and two mitochondrial gene regions (*mtMutS*, *igr1 + COI*) were sequenced using previously published primers and protocols (Alderslade and McFadden 2007) (GenBank accession nos. KY979504, KY979505). Attempts to sequence the nuclear 28S rDNA gene failed. *mtMutS* (699 bp) and *COI* (coding region only, 560 bp) were aligned with the comprehensive, 130-taxon dataset of McFadden and Ofwegen (2012), which includes representatives of all other sclerite-free octocoral taxa for which molecular data are available: *Acanthoaxis wirtzi*, *Acrossota amboinensis*, *Cervera atlantica* (Johnson, 1861), *Cornularia cornucopiae* and *C. pabloi*, *Malacacanthus capensis*, *Phenganax parrini*, *Xenia hicksoni*, and an undescribed species of *Xenia* from Indonesia (*Xenia* sp. 3, see: McFadden et al. 2014). Phylogenetic analyses followed the procedure of McFadden and Ofwegen (2012) using a combined dataset with different models of evolution applied to two separate data partitions, mitochondrial genes (*mtMutS + COI*; TVM+I+G) and 28S rDNA (GTR+I+G). Bayesian analyses were run using MrBayes v.3.2 (Ronquist et al. 2012) with a GTR+I+G model applied to both data partitions separately. Analyses were run for 4 million generations (until standard deviation of split partitions <0.01) with a burn-in of 25% and default Metropolis coupling parameters. Pairwise genetic distance values (Kimura 2-parameter) between species were estimated using MEGA v. 5.10 (Tamura et al. 2011). Alignment and tree files are available in TreeBase (http://purl.org/phylo/treebase/phylows/study/TB2:S20950).

## Results

### Systematic section

#### Family Clavulariidae Hickson, 1894

##### Sub-family Clavulariinae Roxas, 1933

###### Altumia gen. n.

Taxon classificationAnimaliaAlcyonaceaClavulariidae

http://zoobank.org/03DD2AAA-D26D-4E41-A6B8-A6DB1CAEF749

####### Diagnosis.

Clavulariinae with a thin and soft encrusting base, sometimes resembling a short stolon. Polyps erect when expanded, separate from each other; the stolon may feature a few polyps, occasionally only one. Polyps fully retractile into base of the colony, forming low truncated dome-shaped mounds. No sclerites in any part of the colony. Colonies lack symbiotic algae (zooxanthellae). Type species: *Altumia delicata* sp. n. by original designation and monotypy.

####### Etymology.

The generic name is derived from the Latin ‘altum’, deep, referring to the habitat of the new genus at MCE depths and beyond. Gender female.

####### Molecular results.

**Figure 1.**
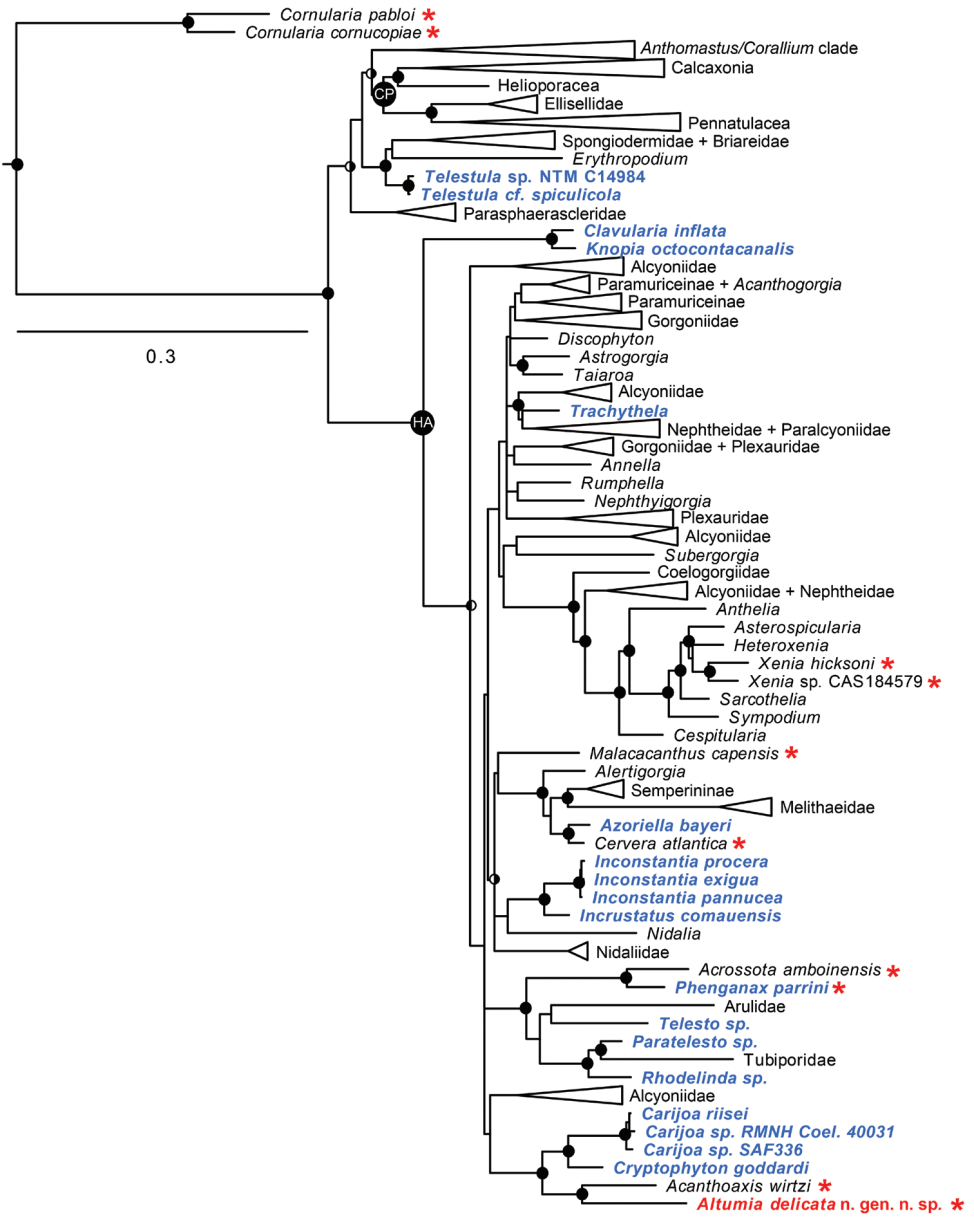
Phylogenetic relationships among species of octocorals that lack sclerites (red asterisks) and members of family Clavulariidae (blue labels), including *Altumia delicata* gen n. sp. n. (red label). Solid circles at nodes indicate strong support from both maximum-likelihood (bootstrap value >70%) and Bayesian (posterior probability >0.90) analyses; split circles indicate strong support from one analysis only (left half solid: supported by ML; right half solid: supported by Bayesian analysis). Strongly supported clades that include no clavulariid or sclerite-free species have been collapsed. Hexacorallian outgroup taxa used to root tree are not shown. For a comprehensive list of taxa and sequences included in the analyses see McFadden and Ofwegen (2012).

Maximum likelihood and Bayesian analyses yielded identical tree topologies that both support the phylogenetic placement of *Altumia* n. gen. as the sister taxon to *Acanthoaxis wirtzi* (Acanthoaxiidae), within a larger well-supported clade that also includes the Clavulariidae genera *Carijoa* F. Müller, 1867 and *Cryptophyton* Williams, 2000 (Figure 1). Pairwise genetic distances (Kimura 2-parameter) between *Altumia* n. gen. and *Acanthoaxis* (*mtMutS*: 9.4%, *COI*: 2.9%) are comparable to values among different genera and some family-level clades of octocorals (McFadden et al. 2011).

###### Altumia delicata sp. n.

Taxon classificationAnimaliaAlcyonaceaClavulariidae

http://zoobank.org/4D9A82B8-F305-43E1-A5A4-3F9DB672781A

Figures 2 , 3

####### Holotype.

ZMTAU CO 37427, Israel, Gulf of Aqaba, Eilat, 29°30'38.31"N, 34°55'59.30"E, 132 m, 30 May 2016, collected by ROV, coll. M. Weis; paratype: ZMTAU CO 37495, Israel, Gulf of Aqaba, Eilat, 29°30'37.29"N, 34°55'59.28"E, 118 m, 8 March 2017, collected by ROV, coll. M. Weis

####### Diagnosis.

**Figure 2.**
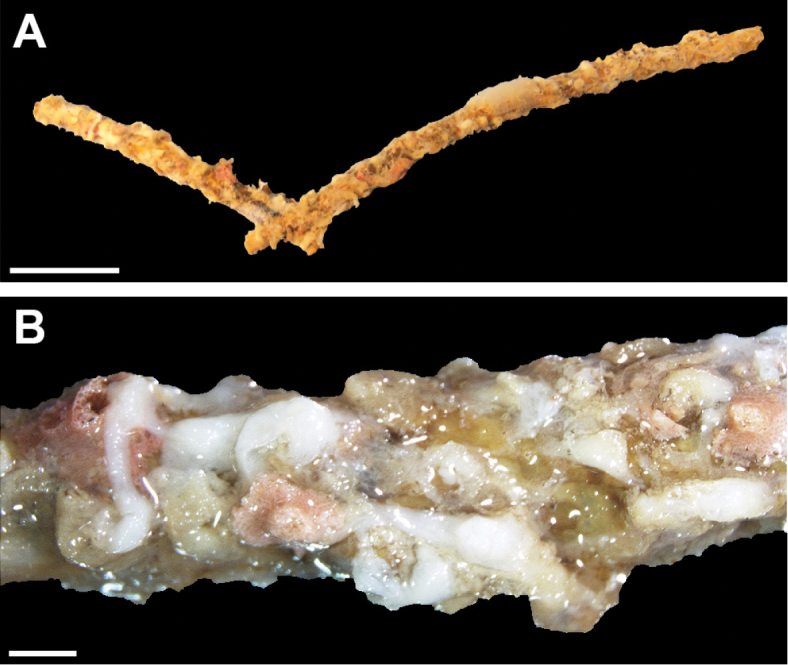
*Altumia delicata* gen. n. sp. n. holotype ZMTAU CO 37427. **A** Colony growing over a branch of a black coral **B** close up of holotype. Scale 10 mm at **A**, 1 mm at **B**.

The ethanol-preserved holotype is comprised of thin patches of short stolon-like crusts growing over the dead branch of a black coral (Antipatharia) (Figure 2A), almost invisible to the naked eye. The milky-white, thin (<0.5 mm) crusts are a few mm long (Figure 2B), very soft, almost slime-like. Polyps completely retracted and practically invisible in the preserved colonies. No sclerites observed in any part of the colony.

**Figure 3.**
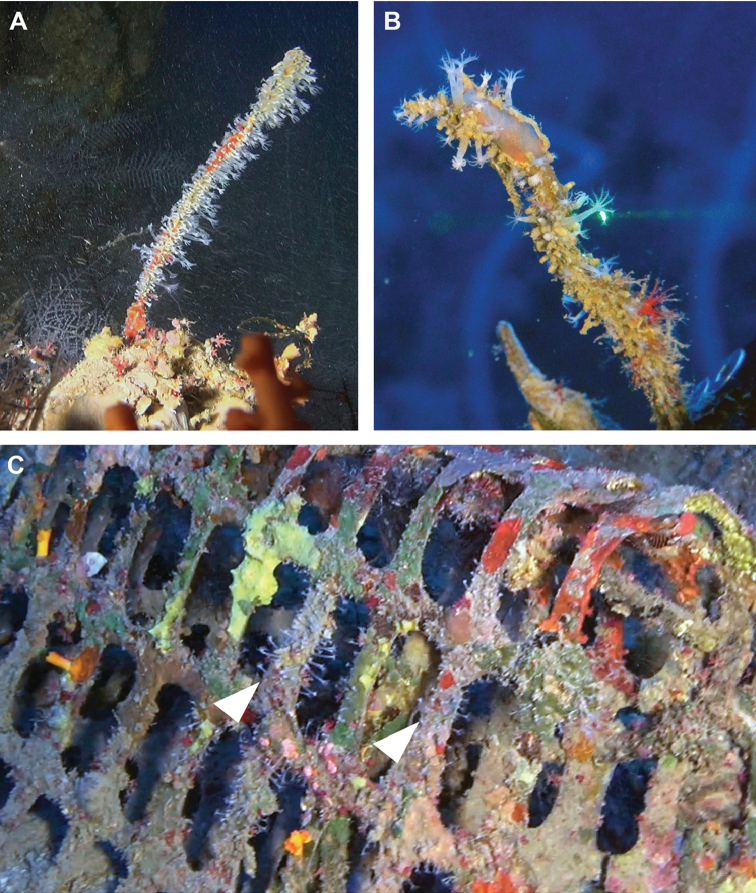
*Altumia delicata* gen. n. sp. n. live colonies. **A, B** colonies growing over branch of black coral with expanded polyps **C** colonies growing on PVC net (arrow heads).

When alive, the delicate, semi-transparent expanded polyps are distinct and are up to 20 mm long, featuring eight pinnate tentacles (Figure 3A). The ROV photographs indicate that the colonies commonly grow on dead black corals; the latter may reach a large size (~45 cm in length) and can be predominantly fouled by *A. delicata* (Figure 3B). Interestingly, debris, such as PVC net found at a depth of 100 m, was found to be colonized by this octocoral (Figure 3C).

####### Intraspecific variability.

There are no differences between the holotype and the paratype except for the size of the colonies.

####### Etymology.

The species name is formed from the Latin ‘delicata’, delicate, referring to the fine texture of the colonies and their polyps. Gender female.

#### Discussion

Assignment of *Altumia* gen. n. to family Clavulariidae is complicated by the recognition that this family is highly polyphyletic, comprising at least seven distinct clades distributed across the Octocorallia (McFadden and Ofwegen 2012). Among other species that lack sclerites, *Altumia delicata* n. gen. n. sp. is most similar morphologically to *Cervera atlantica*, an azooxanthellate, stoloniferous species formally classified in the family Cornulariidae (López-González and Garcia-Gomez 1995). *Cervera* is, however, far removed phylogenetically from *Cornularia*, a genus that is characterized by a theca-like peridermal envelope that surrounds the polyp, a unique morphological feature not found in *Cervera* or any other octocoral. Phylogenetically, *Cornularia* occupies a unique, basal position within Octocorallia. In contrast, *Cervera* belongs to the large Holaxonia-Alcyoniina clade, where it is a close sister taxon of *Azoriella*, a genus of Clavulariidae (Figure 1). These morphological and phylogenetic differences suggest that *Cervera* should not be classified in Cornulariidae, but instead in Clavulariidae. *Cervera* and *Altumia* gen. n. are also morphologically similar to another clavulariid, *Phenganax parrini*, although that sclerite-free species has zooxanthellae.

Despite its morphological similarity to *Cervera* and *Phenganax*, the molecular phylogenetic analyses suggest that *Altumia* gen. n. is not closely related to either of those genera (Figure 1). Instead it falls within a clade comprising two other genera of Clavulariidae, *Carijoa* and *Cryptophyton*, as well as *Acanthoaxis wirtzi*, the sole member of family Acanthoaxiidae. *Altumia* gen. n. is, however, morphologically distinct from each of those genera. *Carijoa* and *Cryptophyton* both have sclerites; *Carijoa* is further distinguished by a growth form in which secondary polyps arise from primary, axial polyps, while *Cryptophyton* has a membranous growth form. Like *Altumia* gen. n., *Acanthoaxis wirtzi* lacks sclerites, but it has an axis of gorgonin with a hollow, cross-chambered core and spines, which are unique morphological features that define the monotypic family Acanthoaxiidae. Because of its morphological similarity to other Clavulariidae and its phylogenetic position within a clade that includes members of that family, we have assigned *Altumia* gen. n. to Clavulariidae. As discussed by McFadden and Ofwegen (2012), the polyphyletic family Clavulariidae will require extensive revision once the morphological distinctions among its phylogenetically distinct, component clades are better understood.

At present there is only scant information on MCE octocorals (Shoham and Benayahu 2017 and references therein), and no study has referred to any octocoral similar to *Altumia delicata*. Undoubtedly, the discovery of *A. delicata* highlights the need for an in-depth study of MCE octocoral diversity. The photographic records from the ROV indicate a continuous distribution of this species from 69 to 140 m depth and, therefore, it can be concluded that it inhabits lower MCEs (> 60 m, see Loya et al. 2016). However, its possible occurrence at deeper sites should be further explored. For instance, Kahng et al. (2014) noted that it is still unclear whether MCEs host specialized coral communities, or are merely marginal extensions of their shallower counterparts.

#### Conclusion

The current study highlights the possibility that MCEs may host octocorals also found below the deepest fringes of these MCEs; and that specifically, deep-water octocorals may populate the zone alongside those of the lower MCEs, contributing to the biodiversity there. Consequently, questions related to the genetic/demographic connectivity between MCEs and shallower reefs (Loya et al. 2016) are potentially relevant to understanding the connectivity between MCEs and deeper communities.

## Supplementary Material

XML Treatment for Altumia

XML Treatment for Altumia delicata
